# Implementation of parallel spatial stochastic reaction-diffusion simulation in STEPS

**DOI:** 10.1186/1471-2202-16-S1-P54

**Published:** 2015-12-18

**Authors:** Weiliang Chen, Iain Hepburn, Erik De Schutter

**Affiliations:** 1 Computational Neuroscience Unit, Okinawa Institute of Science and Technology, Okinawa 904-0411, Japan; 2 Theoretical Neurobiology, University of Antwerp, B-2610 Antwerpen, Belgium

## 

Spatial stochastic reaction-diffusion simulation has been recognized as an essential modeling tool in computational neuroscience studies of signaling pathways, as shown in an increasing number of recent studies such as our previous work on the stochastic effects of calcium dynamics in Purkinje cells [1]. A critical performance issue arises when attempting to model large pathway models with complex morphologies, for example the one proposed in Human Brain Project [2], due to the serial nature of Gillespie's direct method [3], the fundamental algorithm of many spatial stochastic reaction-diffusion simulators including STEPS [4]. Various solutions have been proposed to improve the computational efficiency of the Gillespie method, including the tau-leaping approximation [5], most of which, however, remain serial implementations.

The need of parallel implementation of stochastic reaction-diffusion simulators has become urgent, as the scale and complexity of model being studied surpasses the speedup gained from hardware upgrade and algorithm improvement. However, such a task is not trivial as the original Gillespie SSA is known to be extremely serial.

In CNS2014 we proposed a parallel solution to approximate diffusion events in STEPS [6], which significantly improves the performance while maintaining high accuracy. We now further improve this solution by introducing a multinomial algorithm for fast diffusion direction selection of multiple molecules in a single subvolume. We also combine this diffusion approximation with a new operator splitting solution for reaction events in the SSA system. The combined solution is implemented in STEPS as its first parallel solver named TetOpSplit. Current implementation of TetOpSplit uses MPI as its parallel protocol and aims to provide solutions for large scale simulations such as whole cell reaction-diffusion models, in modern supercomputers like Blue Gene.

In this poster we discuss the difficulties we encountered during the transformation from the serial TetOpSplit algorithm to its parallel counterpart, as well as our solutions. We also provide performance results of the new solver via different examples, and compare them with the results gained from our original serial SSA implementation.

## References

[B1] Anwar H Hepburn I Nedelescu H Chen W De Schutter E Stochastic Calcium Mechanisms Cause Dendritic Calcium Spike Variability J Neurosci 2013 33 40 15488 15867 24089492 10.1523/JNEUROSCI.1722-13.2013 PMC6618479

[B2] The Human Brain Project http://www.humanbrainproject.eu

[B3] Gillespie DT Exact Stochastic Simulation of Coupled Chemical Reactions The Journal of Physical Chemistry 1977 81 25 2340 2361

[B4] Hepburn I Chen W Wils S De Schutter E STEPS: efficient simulation of stochastic reaction-diffusion models in realistic geometries BMC Syst Biol 2012 6 36 22574658 10.1186/1752-0509-6-36 PMC3472240

[B5] Gillespie DT Approximate accelerated stochastic simulation of chemically reacting systems" The Journal of Chemical Physics 2001 115 4 1716 1711

[B6] Hepburn I Accurate approximation and MPI parallelization of spatial stochastic reaction-diffusion in STEPS BMC Neuroscience 2014 15 Suppl 1 P177

